# Letter to the Editor: Assay-specific Spurious ACTH Results Lead to Misdiagnosis, Unnecessary Testing, and Surgical Misadventure—A Case Series

**DOI:** 10.1210/jendso/bvz011

**Published:** 2019-11-27

**Authors:** Ghaith Altawallbeh, Amy B Karger

**Affiliations:** Department of Laboratory Medicine and Pathology, University of Minnesota, Minneapolis, Minnesota

In an article recently published in the *Journal of the Endocrine Society*, Greene et al. (1) describe 5 cases collected over 7 years with falsely elevated results with the Siemens Immulite adrenocorticotropic hormone ACTH assay, which had accurate results with the Roche Cobas or Tosoh AIA ACTH assays. Based on this, the authors discourage use of the Siemens Immulite assay and conclude: “We believe that an alternate ACTH assay such as the ACTH (Cobas) or ACTH (AIA) should be used in the diagnosis and differential diagnosis of patients with suspected disorders of pituitary–adrenal function” (1). While we agree that it is important to make readers aware of rare issues with erroneous laboratory results, we disagree with the authors’ conclusions.

It is important to recognize that the alternate ACTH methods on the Roche Cobas or Tosoh AIA instruments are also immunoassays, and thus also susceptible to rare erroneous results. Since the authors’ main purpose was to educate endocrinologists about rare erroneous results with the Siemens Immulite ACTH assay, it is obligatory for them to acknowledge that the other immunoassay methods they suggest as replacements for the Siemens Immulite assay are also subject to interferences. For example, Morita et al. (2) reported a case where heterophilic antibodies interfered with the Roche Cobas Elecsys ACTH assay. In this case report, both the Siemens Immulite and the Tosoh assay provided accurate results consistent with the patient’s clinical status. An additional study by Toprak et al. (3) described a falsely low ACTH result due to EDTA interference observed with the Roche Cobas Elecsys ACTH assay. In this study a 2-fold and 4-fold increase in EDTA concentration, which can occur with underfilled sample tubes, resulted in a 19% and 50% decrease in ACTH results, respectively. For the Tosoh AIA ACTH assay, an urgent medical device recall notice was issued on November 30, 2018, informing end users that fluorescein angiography procedures can cause interferences with their ACTH assay, recommending use of an alternate method in these cases (4). Lastly, querying the term “ACTH” in the online Food and Drink Administration (FDA) Manufacturer and User Facility Device Experience (MAUDE) database, which houses medical device reports submitted to the FDA, reveals reports of erroneous ACTH results on multiple immunoassay instruments (5).

In conclusion, all FDA-approved ACTH assays are immunoassays, and all may be susceptible to rare interferences causing false results. Switching from one immunoassay to another will not prevent the occurrence of rare erroneous results, and there is no evidence that the Siemens Immulite is more prone to interferences than other ACTH immunoassays. Therefore we disagree with the authors’ recommendation to discontinue use of the Siemens Immulite ACTH assay in favor of other ACTH assays. Rather, providers need to be educated that false results can occur with any immunoassay, and should be advised that if a patient’s result is inconsistent with other results or does not fit the clinical picture, they should contact the clinical laboratory for further troubleshooting and investigation.

## Abbreviations

ACTH: adrenocorticotropic hormone
FDA: Food and Drink Administration

## Additional Information

*Disclosure Summary:* Dr. Karger receives research and salary support from Siemens Healthcare Diagnostics for collaborative studies unrelated to the content of this publication.

## References

[1] Greene LW, Geer EB, Page-Wilson G, Findling JW, Raff H Assay-Specific Spurious ACTH Results Lead to Misdiagnosis, Unnecessary Testing, and Surgical Misadventure-A Case Series. J Endocr Soc. 2019;3(4):763–772. 30963134 10.1210/js.2019-00027 PMC6446888

[2] Morita K, Ogawa M, Kimura M, et al Falsely elevated plasma ACTH levels measured by the Elecsys assay related to heterophilic antibody in a case of secondary adrenocortical insufficiency. Endocr J. 2019;66(6):563–569. 30944263 10.1507/endocrj.EJ19-0023

[3] Toprak B, Yalcin H, Arı E, Colak A EDTA interference in electrochemiluminescence ACTH assay. Ann Clin Biochem. 2016;53(6):699–701. 27166315 10.1177/0004563216636898

[4] U.S. Food and Drug Administration Medical Devices Databases, Class 2 Device Recall ST AIAPACK ACTH (ACTH). https://www.accessdata.fda.gov/scripts/cdrh/cfdocs/cfRes/res.cfm?ID=169038. Accessed October 21, 2019.

[5] U.S. Food and Drug Administration Manufacturer and User Facility Device Experience Database. https://www.accessdata.fda.gov/scripts/cdrh/cfdocs/cfMAUDE/TextSearch.cfm. Accessed October 21, 2019.

